# Impact of Simultaneous Initiation of Finerenone and Empagliflozin on Urinary Albumin-to-Creatinine Ratio in Asia: Pre-Specified Analysis of CONFIDENCE

**DOI:** 10.2215/CJN.0000000865

**Published:** 2025-09-18

**Authors:** Rajiv Agarwal, Jennifer B. Green, Hiddo J.L. Heerspink, Johannes F.E. Mann, Janet B. McGill, Amy Mottl, Takeshi Osonoi, Atanu Pal, Peter Rossing, Julio Rosenstock, Muthiah Vaduganathan, Li Li, Na Li, Charlie Scott, Pravin Manjrekar, Satoshi Yamashita, Masaomi Nangaku

**Affiliations:** 1 Richard L. Roudebush VA Medical Center, Indiana University School of Medicine, Indianapolis, Indiana; 2 Duke University School of Medicine, Durham, North Carolina; 3 Department of Clinical Pharmacy and Pharmacology, University of Groningen, Groningen, The Netherlands; 4 KfH Kidney Centre Munich, Friedrich Alexander University, Munchen, Germany; 5 Division of Endocrinology, Metabolism & Lipid Research, Washington University in St. Louis, St. Louis, Missouri; 6 University of North Carolina School of Medicine, Chapel Hill, North Carolina; 7 Naka Kinen Clinic, Ibaraki, Japan; 8 Institute of Post Graduate Medical Education and Research and SSKM, Kolkata, India; 9 Steno Diabetes Center, University of Copenhagen, Copenhagen, Denmark; 10 Velocity Clinical Research at Medical City, Dallas, Texas; 11 Brigham and Women's Hospital and Harvard Medical School, Boston, Massachusetts; 12 Bayer AG, Berlin, Germany; 13 Bayer Healthcare, Beijing, China; 14 Bayer Healthcare Inc, Whippany, New Jersey; 15 Bayer Pharmaceuticals, Mumbai, Maharashtra, India; 16 Bayer Yakuhin Ltd, Osaka, Japan; 17 The University of Tokyo Graduate School of Medicine, Tokyo, Japan

**Keywords:** CKD

## Abstract

**Key Points:**

- Finerenone plus empagliflozin was more effective in reducing urinary albumin-to-creatinine ratio than either drug alone in Asian participants of the CONFIDENCE trial.
- Except for hyperkalemia, adverse events and serious adverse events were less frequent in participants from Asia than in the overall CONFIDENCE trial population.
- The risk–benefit profile of the combination seems similarly favorable in people from Asia as in the overall CONFIDENCE trial population.

**Background:**

The COmbinatioN effect of FInerenone anD EmpaglifloziN in participants with CKD and type 2 diabetes using a urinary albumin-to-creatinine ratio (UACR) Endpoint (CONFIDENCE) trial compared simultaneous initiation of finerenone with empagliflozin versus either drug alone. We report a pre-specified participant-level exploratory analysis of outcomes in the subpopulation from Asia (India, Japan, the Republic of Korea, and Taiwan).

**Methods:**

Adults with CKD and type 2 diabetes with UACR between 100 and 5000 mg/g and on a renin–angiotensin system inhibitor were recruited. Participants were randomized 1:1:1 to 180 days of treatment with finerenone 10/20 mg once daily (and empagliflozin-matching placebo), empagliflozin 10 mg once daily (and finerenone-matching placebo), or a combination of both. The primary efficacy outcome was change from baseline in UACR at 180 days. Safety and tolerability were also assessed.

**Results:**

This analysis included 360 participants from Asia. At day 180, the reduction in UACR with combination therapy was 30% greater than that with finerenone alone (95% confidence interval [CI], 12% to 45%; *P* = 0.003) and 34% greater than that with empagliflozin alone (95% CI, 16% to 47%; *P* < 0.001). In general, adverse and serious adverse events were numerically less common in participants from Asia than in those from Europe/North America, except for hyperkalemia. Investigator-reported (10% versus 7%) and laboratory-assessed hyperkalemia (serum potassium >5.5 mmol/L; 19% versus 11%) were more frequent in participants from Asia; however, eGFR declines of >30% at day 30 were less frequent in Asia (3% versus 5%).

**Conclusions:**

Simultaneous initiation of finerenone and empagliflozin was effective and well tolerated among CONFIDENCE study participants from Asia, consistent with the overall population.

## Visual Abstract

**Figure:**
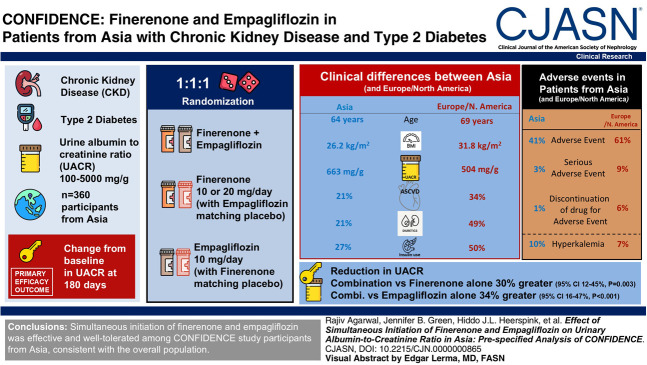

## Introduction

Of the approximately eight billion people on the planet, 62% reside in Asia.^1^ The number of people in Asia with type 2 diabetes is increasing.^2,3^ More than 60% of all people with diabetes live in Asia, with almost one-half in China and India combined.^4^ CKD is a common complication of type 2 diabetes^5^ and is therefore expected to grow among Asian people and is projected to increase in the absence of interventions.^6^ The prevalence, morbidity, and mortality of CKD associated with type 2 diabetes are high in parts of the world with low sociodemographic index.^7^ Among population groups, substantial variation can exist in diet, activity levels, environment, genetics, and anthropometric factors.^8–11^ These factors contribute to heterogeneity of disease risk; furthermore, treatment outcomes may also be affected by ethnogeographic differences in pharmacokinetics and pharmacodynamics.^8–12^ Although recent literature emphasizes the need for greater diversity in clinical trial populations and more comprehensive reporting of population characteristics to better understand and generalize treatment effects across diverse patient groups,^13^ the proportions of Asian people in randomized controlled trials are often small.^14–16^

Additive treatment effects are possible with coadministration of finerenone, a nonsteroidal mineralocorticoid receptor antagonist, with a sodium–glucose cotransporter 2 inhibitor (SGLT2i).^17^ The COmbinatioN effect of FInerenone anD EmpaglifloziN in participants with CKD and type 2 diabetes using a urinary albumin-to-creatinine ratio (UACR) Endpoint (CONFIDENCE) trial demonstrated that simultaneous initiation of finerenone with the SGLT2i empagliflozin was superior to either drug alone on a background of angiotensin-converting enzyme inhibitor (ACEi) or angiotensin receptor blocker (ARB) therapy in reducing albuminuria at 180 days.^17^ Simultaneous initiation of finerenone plus empagliflozin led to a greater reduction in UACR compared with finerenone or empagliflozin monotherapy.^17^ The trial was conducted in a large cohort of participants spread across Asia, Europe, and North America.^17^ The primary analysis showed that at day 180, the reduction in UACR with combination therapy was significantly greater than with finerenone or empagliflozin alone (differences of 29% and 32%, respectively; *P* < 0.001 for both comparisons).^17^ Subgroup analysis reported in the primary publication showed consistency in the primary efficacy outcome between all regions of the study.^17^

Here, we report a pre-specified participant-level exploratory subgroup analysis among CONFIDENCE participants from Asia, who constituted 46% of the overall study population. As in the main analysis, the safety and efficacy of the combination therapy were compared with either drug alone.

## Methods

### Study Design

The CONFIDENCE trial (NCT05254002) was a Phase 2, double-blind, randomized, three-arm study assessing the efficacy and safety of finerenone plus empagliflozin versus either finerenone or empagliflozin alone in people with CKD and type 2 diabetes. The trial design and baseline characteristics have been published previously^17,18^ and will be described in brief here. The trial was conducted across 14 countries/regions, of which four were in Asia (India, Japan, the Republic of Korea, and Taiwan); the remaining 10 countries were in Europe and North America. The trial was approved by the institutional review boards at each study site. The trial was conducted in accordance with the principles of the Declaration of Helsinki. All participants provided written informed consent.

### Participants

This analysis reports a subgroup from Asia. Individuals aged at least 18 years with type 2 diabetes (glycated hemoglobin [HbA1c] <11%), eGFR 30–90 ml/min per 1.73 m^2^, and albuminuria (UACR between 100 and 5000 mg/g) were enrolled. Exclusion criteria included type 1 diabetes and serum potassium >4.8 mmol/L. All participants were required to be receiving the clinically maximum tolerated dose of a renin–angiotensin system inhibitor (ACEi or ARB) for more than 1 month at the screening visit and must not have taken an SGLT2i or potassium binder agent within the 8 weeks before screening.

### Procedures

Participants were randomly assigned in a 1:1:1 ratio to receive finerenone 10 or 20 mg once daily (and empagliflozin-matching placebo), empagliflozin 10 mg once daily (and finerenone-matching placebo), or a combination of the two.

The duration of study treatment was 180 days. Safety and efficacy assessments were performed during clinic visits scheduled 14, 30, 90, 180, and 210 days after the start of treatment. Permitted concomitant therapies included insulin, insulin secretagogues, thiazolidinediones, ACEis, ARBs, loop diuretics, thiazides, and glucagon-like peptide-1 receptor agonists (GLP-1 RAs).

Urine and blood specimens were collected at clinic visits throughout the study for the assessment of UACR, serum potassium, and eGFR. BP was also measured at every clinic visit. Adverse events (AEs) were coded using the Medical Dictionary for Regulatory Affairs.

### Outcomes

The primary efficacy outcome was the change in log-transformed mean UACR from baseline to 180 days. Secondary efficacy outcomes included change in UACR from baseline to 30 days after the end-of-treatment visit as well as the proportion of participants achieving a reduction in UACR of more than 30%, 40%, and 50% at 180 days. Secondary safety outcomes included changes from baseline in eGFR, serum potassium, and systolic BP as well as AEs, laboratory data, vital signs, and hyperkalemia events.

### Statistical Analysis

Efficacy analyses were performed using the full analysis set (FAS), which comprised all randomized participants, except for those who were misrandomized and did not take study treatment and those in whom Good Clinical Practice was not followed correctly. A mixed model for repeated measures, with the factors treatment group, visit, treatment by visit interaction, factors for the two stratification levels (UACR category and eGFR category), log-transformed UACR baseline value, and log-transformed UACR baseline value by visit interaction, was used for the primary efficacy outcome.^17^ The combination therapy group was compared with the monotherapy groups by calculating percentage differences and two-sided 95% confidence intervals (CIs), derived from least-squares (LS) mean pairwise ratios. The CKD Epidemiology Collaboration equation was used to calculate eGFR values,^19^ and a modified version of the equation was used for participants from Japan.^20^ Changes in eGFR, serum potassium, and systolic BP were analyzed using similar methods as in the primary efficacy outcome, with calculation of LS means using a mixed model for repeated measures. Safety analyses were conducted in the safety analysis set, comprising all randomized participants who took at least one dose of study treatment.

## Results

### Participants

**Figure 1:**
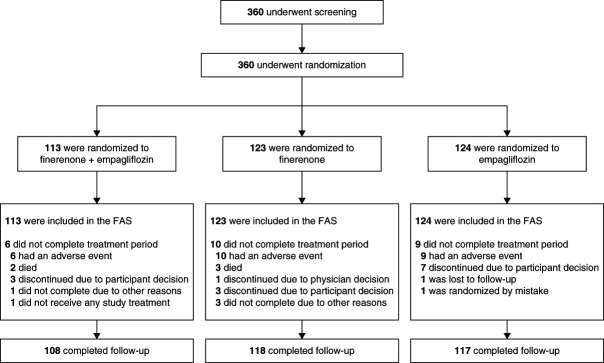
**CONSORT diagram: participants from Asia.** FAS, full analysis set; GCP, good clinical practice.

In the CONFIDENCE study, a total of 1664 participants underwent screening from June 23, 2022, to August 14, 2024, and 800 individuals were randomized to treatment and comprised the FAS.^17^ In Asia, 360 of 708 candidates who underwent screening were included in the FAS (Figure 1): 132 (37%) in India, 76 (21%) in Japan, 104 (29%) in the Republic of Korea, and 48 (13%) in Taiwan (Table 1). The Asian population was randomized to receive study treatment as follows: combination therapy (*n*=113), finerenone (*n*=123), or empagliflozin (*n*=124). As presented in Table 1, baseline characteristics of Asian participants were consistent across the three study groups. The mean (±SD) age was 64±11 years, the mean (±SD) eGFR was 53±17 mL/min per 1.73 m^2^, and the mean (±SD) HbA1c was 7.3%±1.2%. All except four participants (356/360, 99%) were taking an ACEi or ARB at baseline.

**Table 1:** Demographic and baseline characteristics in participants from Asia

Baseline Characteristics	Finerenone+empagliflozin	Finerenone	Empagliflozin	Total
*N*, (%)	113 (31)	123 (34)	124 (34)	360 (100.0)
Age, yr, mean±SD	66±11	63±10	63±10	64±11
Men, *n* (%)	91 (80.5)	100 (81.3)	95 (76.6)	286 (79.4)
**Country, *n* (%)**				
India	34 (30)	53 (43)	45 (36)	132 (37)
Japan	32 (28)	20 (16)	24 (19)	76 (21)
Republic of Korea	34 (30)	33 (27)	37 (30)	104 (29)
Taiwan	13 (12)	17 (14)	18 (15)	48 (13)
Baseline eGFR values,^a^ ml/min per 1.73 m^2^, mean±SD	51±16	54±18	53±16	53±17
UACR, mg/g, median (IQR)	645 (336–1003)	738 (292–1653)	654 (362–1233)	663 (327–1291)
**Severity of albuminuria, *n* (%)**				
<300 mg/g	21 (19)	31 (25)	26 (21)	78 (22)
300 to <1000 mg/g	63 (56)	41 (33)	60 (48)	164 (46)
≥1000 mg/g	28 (25)	50 (41)	38 (31)	116 (32)
ASCVD,^b^ *n* (%)	23 (20)	24 (20)	30 (24)	77 (21)
Weight, kg, mean±SD	68.8±12.7	71.6±14.2	70.6±12.7	70.4±13.2
Height, cm, mean±SD	163.2±8.3	164.4±7.6	163.9±8.9	163.9±8.3
BMI, kg/m^2^, mean±SD	25.8±3.8	26.4±4.2	26.3±4.1	26.2±4.0
Systolic BP, mm Hg, mean±SD	132.7±14.0	132.4±11.9	133.3±12.0	132.8±12.6
Serum potassium value, mmol/L, mean±SD	4.5±0.4	4.5±0.5	4.6±0.4	4.5±0.4
HbA1c, %, mean±SD	7.2±1.2	7.3±1.3	7.3±1.2	7.3±1.2
**Concomitant medications, *n* (%)**				
Insulin	25 (22)	31 (25)	42 (34)	98 (27)
Metformin	70 (62)	78 (63)	78 (63)	226 (63)
GLP-1 RA	15 (13)	11 (9)	14 (11)	40 (11)
DPP4 inhibitors	64 (57)	70 (57)	70 (57)	204 (57)
Insulin secretagogues	57 (50)	48 (39)	32 (26)	137 (38)
ACEis or ARBs	113 (100)	122 (99)	121 (98)	356 (99)
Antihypertensives	113 (100)	122 (99)	122 (98)	357 (99)
*β*-blockers	29 (26)	37 (30)	27 (22)	93 (26)
Calcium channel blockers	81 (72)	82 (67)	80 (65)	243 (68)
Diuretics	26 (23)	31 (25)	17 (14)	74 (21)
Antiplatelets	36 (32)	37 (30)	35 (28)	108 (30)
Statins	75 (66)	81 (66)	72 (58)	228 (63)

ACEi, angiotensin-converting enzyme inhibitor; ARB, angiotensin receptor blocker; ASCVD, atherosclerotic cardiovascular disease; BMI, body mass index; DPP4, dipeptidyl peptidase-4; GLP-1 RA, glucagon-like peptide-1 receptor agonist; HbA1c, glycated hemoglobin; IQR, interquartile range; UACR, urinary albumin-to-creatinine ratio.

a The eGFR was calculated with the use of the CKD Epidemiology Collaboration equation, which was modified for the Japanese participants.

b Coded using MedDRA version 27.0.

Supplemental Table 1 presents baseline characteristics among participants from Asia versus Europe/North America. Those from Asia were younger (mean age 64 versus 69 years), had a lower mean body wt (70.4 versus 91.4 kg), lower mean body mass index (26.2 versus 31.8 kg/m^2^), lower mean systolic BP (133 versus 137 mm Hg), higher median UACR (663 versus 504 mg/g), and a greater proportion had ≥1000 mg/g albuminuria (32% versus 23%). Those from Asia had less atherosclerotic cardiovascular disease (ASCVD; 21% versus 34%), while the mean HbA1c level was similar in participants from Asia and Europe/North America. Percentages of participants using statins (63% versus 84%) and antiplatelet agents (30% versus 48%) were lower in those from Asia versus Europe/North America. The pattern of antidiabetic drug use was different in that participants from Asia had less insulin and GLP-1 RA use but greater use of dipeptidyl peptidase-4 inhibitors and insulin secretagogues such as sulfonylureas. Furthermore, the pattern of antihypertensive agent use was also different, with less diuretic and *β*-blocker use, but greater calcium channel blocker use observed in participants from Asia compared with those from Europe/North America.

### Primary End Point

The median (interquartile range) UACR at baseline in Asian participants was 645 (336–1003) mg/g in the combination therapy group, 738 (292–1653) mg/g in the finerenone group, and 654 (362–1233) mg/g in the empagliflozin group. At 180 days after randomization, UACR data were available for 333 Asian participants (Table 2).

**Table 2:** Changes from baseline in urinary albumin-to-creatinine ratio at 180 days (end of study treatment) and at 210 days (30 days after discontinuation of study treatment) in participants from Asia and Europe/North America

Time Point	Empagliflozin+finerenone (% [95% CI])	Finerenone (% [95% CI])	Empagliflozin (% [95% CI])	Difference between combination therapy and finerenone alone (% [95% CI])	Difference between combination therapy and empagliflozin alone (% [95% CI])
**Asia**					
Day 180	*n=*105 −53 (−60 to −44.2)	*n*=114 −33 (−43 to −21)	*n*=114 −29 (−40 to −17)	−30 (−45 to −12) *P* = 0.003	−34 (−47 to −16) *P* < 0.001
Day 210	*n*=105 −22 (−34 to −9.3)	*n*=108 −11 (−24 to 3)	*n*=112 −14 (−26 to −0.1)	−12 (−29 to 9)	−10 (−27 to 12)
**Europe/North America**					
Day 180	*n*=134 −56 (−62 to −49)	*n*=122 −39 (−47 to −28)	*n*=124 −37 (−46 to −26)	−29 (−43 to −12) *P* = 0.002	−31 (−44 to−15) *P* < 0.001
Day 210	*n*=132 −24 (−33 to −13)	*n*=118 −16 (−27 to −4)	*n*=120 −16 (−27 to −3)	−9 (−25 to 10)	−10 (−25 to 10)

Threshold of UACR reduction	Treatment	Responders/Total	Odds ratio of combination compared to monotherapy (95% CI)	*P*-interaction^a^
**>30% reduction**				
Asia	Combination	77/106		
Asia	Finerenone	54/114	3.02 (1.71 to 5.31)	
Asia	Empagliflozin	56/114	2.84 (1.61 to 5.00)	
Europe/North America	Combination	91/134		0.36
Europe/North America	Finerenone	69/122	1.63 (0.98 to 2.72)	
Europe/North America	Empagliflozin	67/124	1.82 (1.09 to 3.02)	
**>40% reduction**				
Asia	Combination	67/106		
Asia	Finerenone	47/114	2.48 (1.44 to 4.27)	
Asia	Empagliflozin	43/114	2.88 (1.66 to 4.99)	
Europe/North America	Combination	87/134		0.36
Europe/North America	Finerenone	57/122	2.11 (1.28 to 3.49)	
Europe/North America	Empagliflozin	60/124	1.97 (1.19 to 3.25)	
**>50% reduction**				
Asia	Combination	53/106		0.36
Asia	Finerenone	37/114	2.11 (1.22 to 3.65)	
Asia	Empagliflozin	30/114	2.85 (1.62 to 5.03)	
Europe/North America	Combination	78/134		0.18
Europe/North America	Finerenone	47/122	2.22 (1.35 to 3.67)	
Europe/North America	Empagliflozin	46/124	2.35 (1.42 to 3.89)	

a *P*-interaction term includes the joint probability of region and region×treatment interaction terms are zero.

CI, confidence interval; LS, least squares; UACR, urinary albumin-to-creatinine ratio.

At day 180, in Asian participants, the reduction from baseline in UACR with combination therapy was 53% (95% CI, 44% to 60%; Table 2). This reduction was 30% greater than that with finerenone alone (95% CI, 12% to 45%; *P* = 0.003) and 34% greater than that with empagliflozin alone (95% CI, 16% to 47%; *P* < 0.001).

At day 180, in European/North American participants the reduction from baseline in UACR with combination therapy was 56% (95% CI, 49% to 62%; Table 2). This reduction was 29% greater than that with finerenone alone (95% CI, 12% to 43%; *P* = 0.002) and 31% greater than that with empagliflozin alone (95% CI, 15% to 44%; *P* < 0.001).

### Secondary Efficacy and Safety End Points

**Figure 2:**
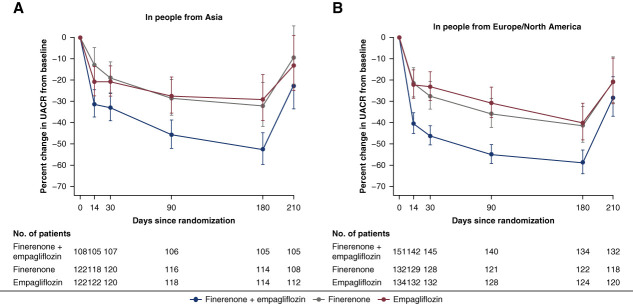
**Change over time in UACR.** Participants from Asia (A) and Europe/North America (B). UACR, urinary albumin-to-creatinine ratio.

Figure 2 shows the change in UACR over time. The UACR among participants in the combination therapy group was reduced by 31% from baseline after 14 days and by 46% from baseline after 90 days. Between the end of treatment and follow-up 30 days later (at day 210), UACR increased in all three study groups (Figure 2 and Table 2). In addition, a greater proportion of participants, both from Asia and Europe/North America, achieved reductions in UACR of >30%, >40%, or >50% with combination therapy versus either finerenone or empagliflozin (Figure 2 and Table 2).

Supplemental Figure 1 shows the LS mean change from baseline in serum potassium level over time in participants from Asia and Europe/North America. The mean serum potassium level increased from baseline during treatment with finerenone plus empagliflozin and returned to near baseline 30 days after the end of study treatment (day 210). In the finerenone group, the mean serum potassium level also increased from baseline during treatment and returned to near baseline at day 210. No major changes from baseline in serum potassium level were observed in the empagliflozin group.

The LS mean change from baseline in systolic BP over time in participants from Asia and Europe/North America is shown in Supplemental Figure 2. The combination of finerenone plus empagliflozin was associated with a reduction in systolic BP from baseline during treatment and a subsequent increase during the 30-day period after treatment discontinuation (from day 180 to day 210). Changes in the systolic BP during the study followed a similar pattern in the finerenone and empagliflozin groups, but the changes were less pronounced than in the combination group.

Supplemental Figure 3 shows the LS mean change from baseline in eGFR over time in participants from Asia and Europe/North America. An early decline in eGFR was observed in the empagliflozin and combination groups, and most of this decline was reversed during the 30 days after drug discontinuation at 180 days. At day 14, the dip in eGFR was less in those from Asia compared with those from Europe/North America.

### AEs

Compared with participants from Europe/North America, overall, those from Asia had numerically lower incidences of AEs (41% versus 61%), treatment discontinuations due to AEs (1% versus 6%), serious AEs (3% versus 9%), and treatment discontinuations due to serious AEs (0% versus 2%). AE incidence rates were similar in all three treatment groups (Table 3). The percentage of participants reporting any AE ranged between 38% and 44% across the three groups, and serious AEs were reported in 2%–4% of participants. One serious AE resulted in the death of a participant (cardiac death in the empagliflozin group); however, no serious AE was treatment-related or led to discontinuation of study treatment.

**Table 3:** Adverse events after treatment initiation in participants from Asia and Europe/North America

Investigator-Reported AEs	Asia				Europe/North America			
	Finerenone+Empagliflozin (*n*=112)	Finerenone (*n*=123)	Empagliflozin (*n*=124)	Total (*N*=359)	Finerenone+Empagliflozin (*n*=156)	Finerenone (*n*=141)	Empagliflozin (*n*=142)	Total (*N*=439)
Any AE, *n* (%)	43 (38)	51 (42)	54 (44)	148 (41)	101 (65)	85 (60)	81 (57)	267 (61)
AE leading to treatment discontinuation, *n* (%)	2 (2)	2 (2)	0 (0)	4 (1)	10 (6)	7 (5)	9 (6)	26 (6)
Any serious AE, *n* (%)	4 (4)	2 (2)	5 (4)	11 (3)	15 (10)	14 (10)	12 (9)	41 (9)
Serious AE leading to treatment discontinuation, *n* (%)	0 (0)	0 (0)	0 (0)	0 (0)	3 (2)	3 (2)	2 (1)	8 (2)
AE with death as the outcome, *n* (%)	0 (0)	0 (0)	1 (0.8)	1 (0.3)	1 (0.6)	0 (0)	1 (0.7)	2 (0.5)
Hyperkalemia,^a^ *n* (%)	12 (11)	17 (14)	6 (5)	35 (10)	13 (8)	13 (9)	4 (3)	30 (7)
Urosepsis or pyelonephritis, *n* (%)	0 (0.0)	0 (0.0)	1 (0.8)	1 (0.3)	1 (0.6)	0 (0.0)	0 (0.0)	1 (0.2)

AE, adverse event.

a The participants with hyperkalemia reported here were those who had received at least one dose of a trial drug and investigator-reported hyperkalemia or blood potassium increased that had started or worsened after the first dose and up to 3 days after any temporary or permanent interruption of the trial treatment.

The proportion of Asian participants with any treatment-emergent hyperkalemia AE was 10% versus 7% for those from outside Asia. In Asia, 11% for those receiving combination therapy, 14% for finerenone alone, and 5% for empagliflozin alone were reported to have hyperkalemia by investigators. No treatment-emergent hyperkalemia AEs led to hospitalization, permanent discontinuation of study drug, or death. Laboratory-assessed hyperkalemia (serum potassium >5.5 mmol/L) was also higher among participants from Asia compared with Europe/North America (19% versus 11%), including mild hyperkalemia (serum potassium >5.5 to ≤6.0 mmol/L; 16% versus 10%) and moderate hyperkalemia (serum potassium >6.0 mmol/L; 6.7% versus 2%; Table 4). By contrast, decline in eGFR of >30% at day 30 was numerically less frequent in participants from Asia compared with those from Europe/North America (3% versus 5%).

**Table 4:** Safety outcomes in participants from Asia and Europe/North America

Safety Assessment	Asia				Europe/North America			
	Finerenone+Empagliflozin	Finerenone	Empagliflozin	Total	Finerenone+Empagliflozin	Finerenone	Empagliflozin	Total
**Serum K level, *n*/*N* (%)**								
>5.5 mmol/L, *n*/*N* (%)	23/111 (21)	32/122 (26)	13/121 (11)	68/354 (19)	17/151 (11)	16/136 (12)	12/136 (9)	45/423 (11)
>5.5 to ≤6.0 mmol/L, *n*/*N* (%)	18/111 (16)	29/122 (24)	11/121 (9)	58/354 (16)	16/151 (11)	14/136 (10)	10/136 (7)	40/423 (10)
>6.0 mmol/L, *n*/*N* (%)	9/112 (8)	11/123 (9)	4/124 (3)	24/359 (7)	3/151 (2)	1/139 (0.7)	3/138 (2)	7/428 (2)
Decline in eGFR^a^ >30% from baseline to day 30, *n*/*N* (%)	6/112 (5)	2/123 (2)	2/124 (2)	10/359 (3)	11/156 (7)	8/141 (6)	1/142 (0.7)	20/439 (5)
Genital mycotic infection, *n*/*N* (%)	0/112 (0.0)	0/123 (0.0)	0/124 (0.0)	0/359 (0.0)	4/156 (3)	0/141 (0.0)	4/142 (3)	8/439 (2)
Symptomatic hypotension, *n*/*N* (%)	0/112 (0.0)	0/123 (0.0)	0/124 (0.0)	0/359 (0.0)	3/156 (2)	0/141 (0.0)	0/142 (0.0)	3/439 (0.7)
AKI, *n*/*N* (%)	0/112 (0.0)	0/123 (0.0)	0/124 (0.0)	0/359 (0.0)	5/156 (3)	3/141 (2)	0/142 (0.0)	8/439 (2)

K, potassium.

a The eGFR was calculated with the use of the CKD Epidemiology Collaboration equation, which was modified for the Japanese participants.

## Discussion

In this exploratory, participant-level subgroup analysis of CONFIDENCE participants in Asia (*n*=360), differences in clinical characteristics were evident compared with those from Europe/North America (*n*=440). Combination therapy with finerenone and empagliflozin was clinically and statistically superior to finerenone or empagliflozin monotherapy in reducing UACR at day 180 among participants from Asia. There was no interaction of geographic distribution—Asia versus Europe/North America—with the benefit of combination therapy compared with monotherapy. Except for hyperkalemia, AE rates were lower in people from Asia than in those from Europe/North America. Indeed, laboratory-assessed hyperkalemia was more frequent in Asia, but acute decline in eGFR was less frequent. AE rates in participants from Asia were comparable across the three study groups, and no hyperkalemia AEs led to discontinuation of study therapy.

The findings of this CONFIDENCE Asia subgroup analysis are consistent with those observed in the overall study population and extend previous findings demonstrating consistency of treatment effect across regions.^17^ Although many baseline characteristics of the Asia subgroup were comparable with those of the overall CONFIDENCE study population, some variations were apparent.^17,21^ These included a younger age, more albuminuria, less ASCVD, and less statin and antiplatelet therapy use in participants from Asia, as well as differences in patterns of antihypertensive and antidiabetic agent use between the Asia subgroup and the overall CONFIDENCE population.

In the primary analysis of the Asia subgroup, the UACR reduction at day 180 with combination therapy was 30% greater than with finerenone alone and 34% greater than with empagliflozin alone. These percentages are similar to the corresponding values in the overall CONFIDENCE population (29% and 32%, respectively).^17^ This degree of similarity demonstrates consistency of treatment effect with combination therapy, despite the variations in participants' baseline characteristics described above and further possible differences not measured in this study (*e*.*g*., genetics, diet, socioeconomic factors). It remains important to continue assessing finerenone and empagliflozin in people from Asia, but these findings suggest that the results of studies performed partly or mainly in other populations may be applicable.

CONFIDENCE evaluated the simultaneous initiation of an SGLT2i in combination with finerenone in people with CKD with albuminuria and type 2 diabetes. In the Finerenone in Chronic Kidney Disease and Type 2 Diabetes: Combined FIDELIO-DKD and FIGARO-DKD Trial Programme Analysis (FIDELITY) series of finerenone studies in people with CKD and type 2 diabetes, finerenone reduced cardiovascular and kidney adverse outcomes irrespective of SGLT2i use at baseline or concomitantly with study medication, without raising any new safety concerns about the combined use of these therapies.^22^ Findings in the FIDELITY participants from Asia were consistent with the overall FIDELITY population.^23^ Finerenone also reduced UACR compared with baseline at 4 months among participants with (37% reduction) or without (31% reduction) SGLT2i use at baseline (*P*_interaction_ = 0.17).^22^ This study confirms that reductions in UACR with finerenone and SGLT2i treatment are independent and additive in Asians people.

As observed with both finerenone^24^ and SGLT2is,^25^ short-term changes in eGFR in response to medication initiation do not correspond with longer term deterioration in kidney function. In other words, a large dip in eGFR over the initial few weeks should not be considered predictive of a large eGFR loss during the following years (also, a long-term reduction may occur in the absence of a large initial decrease). Furthermore, these changes are largely reversible, as the eGFR tends to return toward baseline after discontinuing finerenone, empagliflozin, or their combination, suggesting that the eGFR changes are hemodynamic rather than due to structural changes in the kidney. However, the initial decrease in UACR with finerenone treatment was reported in a prior mediation analysis to mediate most (84%) of the treatment effect of this drug on clinical kidney outcomes, including worsening of eGFR.^26^ In addition, changes in UACR and systolic BP jointly appear to mediate 50% of the cardiovascular effects of finerenone.^27^ With empagliflozin, a prior mediation analysis showed that a combination of hematocrit, HbA1c, systolic BP, and free fatty acids mediated most (79%) of the treatment effect on a composite kidney outcome.^28^

Considering the high incidence of cardiovascular and kidney morbidity in people with type 2 diabetes and CKD, combined use of finerenone and empagliflozin may enable significant mitigation of the disease burden. This may be particularly important for Asia, since this continent has exhibited a woeful increase in the number of people living with diabetes. The large global burden of type 2 diabetes and CKD supports the rationale for routine adoption of comprehensive guideline-based medical therapy, including simultaneous initiation of combination therapy as assessed in CONFIDENCE, to improve clinical outcomes.

Measuring UACR annually in people with type 2 diabetes, as recommended by diabetes and CKD guidelines, can help identify those with kidney disease.^29,30^ More frequent monitoring of UACR and systolic BP after starting treatment for type 2 diabetes can show treatment effects and help encourage adherence with therapy. These measures could foster treatment adherence and help preserve eGFR, thereby optimizing kidney and cardiovascular outcomes in this high-risk population.

Early detection of reductions in systolic BP through home BP monitoring enables timely adjustment of diuretic or dihydropyridine calcium channel blocker doses, helping to prevent hypotension, volume depletion, and related AEs, such as a decline in eGFR that can trigger cessation of combination therapy. This approach allows clinicians to reduce or delay doses when necessary, minimizing the risk of falls, acute decline in eGFR, and other complications, especially in frail populations.

This analysis has shown that the incidence of most AEs in CONFIDENCE participants from Asia were numerically lower than those in participants from Europe/North America. The reduction in the Asian subgroup versus participants from Europe/North America was approximately 50% for any treatment-related AE, and greater reductions were apparent in the rates of AEs leading to discontinuation and rates of serious AEs. The reasons for these differences are unclear, but there are several possibilities. Compared with Europe/North America, people from Asia were younger, had less ASCVD, a lower body mass index, and lower systolic BP. Despite having a similar HbA1c, they required less insulin, suggesting less severe diabetes. Furthermore, cultural differences affecting participants' willingness to report AEs may have contributed. In contrast to the pattern in rates for overall AEs, hyperkalemia was reported more frequently by the investigators in Asia, and this was corroborated by more frequent hyperkalemia recorded by central laboratory monitoring. The incidence of hyperkalemia AEs was also more frequent in the Asia subgroup compared with participants from non-Asian countries in FIDELITY, although in FIDELITY, there was no difference between the Asia and non-Asia subgroups in the frequency of serum potassium >5.5 mmol/L or >6.0 mmol/L.^23^ CONFIDENCE results in participants from Asia and Europe/North America showed that the incidence of hyperkalemia AEs was lower with finerenone plus empagliflozin than with finerenone alone. This mitigation of hyperkalemia is consistent with a prior meta-analysis that reported a reduced risk of severe hyperkalemia with SGLT2is, including in participants treated with a mineralocorticoid receptor antagonist at baseline.^31^ In patients with CKD, certain potassium-rich foods common in Asian diets—such as potatoes, pulses, and green/yellow vegetables—may weakly increase serum potassium, but the overall association between dietary potassium and hyperkalemia is limited, and the bioavailability of potassium from whole foods is often lower than from processed foods or salt substitutes.^32,33^ Other factors such as higher albuminuria and lower use of diuretics are risk factors for hyperkalemia and may be more important than ethnicity.^34,35^ Concomitant use of diuretics was lower in participants from Asia (21%) compared with Europe/North America (49%), which may have contributed to the higher hyperkalemia rate, as well as the lower initial eGFR decline, in the Asian subpopulation. Kidney Disease: Improving Global Outcomes has published global guidance on the evaluation and management of potassium levels in people with kidney disease, although this guidance does not include specific considerations for the Asian population.^36^

Key strengths of this study are the large number of participants from Asia, the inclusion of multiple countries from this region, and the randomized, double-blind design. Limitations include the lack of genetic or socioeconomic profiling of participants (for comparison with the overall study population), and the inherent limitations of subgroup analyses not powered statistically to measure treatment effects in the Asian subpopulation. We also recognize the potential for heterogeneity of results from different countries or regions within Asia, including between the four countries included in our Asian population. Unfortunately, the sample size of the CONFIDENCE study was not sufficient for meaningful assessment of differences at this level.

In conclusion, this study has shown that combination therapy with finerenone and empagliflozin was more effective than either treatment alone in reducing UACR among people from Asia with CKD and type 2 diabetes. The risk–benefit profile of this treatment seems to be similarly favorable in people from Asia as in the overall international population of participants in the CONFIDENCE study. AEs and serious AEs in participants from Asia were fewer, and the risk of hyperkalemia may be related to clinical factors rather than the geographic origins of the participants.

## Supplementary Material

## Data Availability

Availability of the data underlying this publication will be determined according to Bayer's commitment to the EFPIA/PhRMA “Principles for responsible clinical trial data sharing”. This pertains to scope, timepoint and process of data access. As such, Bayer commits to sharing upon request from qualified scientific and medical researchers, patient-level clinical trial data, study-level clinical trial data, and protocols from clinical trials in patients for medicines and indications approved in the United States and European Union as necessary for conducting legitimate research. This applies to data on new medicines and indications that have been approved by the European Union and United States regulatory agencies on or after January 01, 2014. Interested researchers can use www.vivli.org to request access to anonymized patient-level data and supporting documents from clinical studies to conduct further research that can help advance medical science or improve patient care. Information on the Bayer criteria for listing studies and other relevant information is provided in the member section of the portal. Data access will be granted to anonymized patient-level data, protocols and clinical study reports after approval by an independent scientific review panel. Bayer is not involved in the decisions made by the independent review panel. Bayer will take all necessary measures to ensure that patient privacy is safeguarded.

## Supplemental Material

Supplementary Table 1. Demographic and baseline characteristics of participants from Asia and Europe/North America.

Supplemental Figure 1. Least-squares mean change from baseline in serum potassium level over time in participants from Asia (A) and Europe/North America (B).

Supplemental Figure 2. Least-squares mean change from baseline in systolic BP over time in participants from Asia (A) and Europe/North America (B).

Supplemental Figure 3. Least-squares mean change from baseline in eGFR over time in participants from Asia (A) and Europe/North America (B).
