# Supplementary material for: Impact of Simultaneous Initiation of Finerenone and Empagliflozin on Urinary Albumin-to-Creatinine Ratio in Asia: Pre-Specified Analysis of CONFIDENCE
Source: Clin J Am Soc Nephrol. 2025 Sep 18;21(1):72–82. doi: 10.2215/CJN.0000000865 (PMC13135046; doi:10.2215/CJN.0000000865)
Supplement: Supplementary file 2 [file cjasn-21-072-s002.pdf]

Supplemental Material

Supplementary Table 1. Demographic and baseline characteristics of participants from Asia and Europe/North America

	Asia				Europe/North America			
	Finerenone + empagliflozin	Finerenone	Empagliflozin	Total	Finerenone + empagliflozin	Finerenone	Empagliflozin	Total
N**,** (%)	113 (31)	123 (34)	124 (34)	360 (100)	156 (36)	141 (32)	143 (33)	440 (100)
**Age,** years, mean±SD	66±11	63±10	63±10	64±11	69±9	68±10	69±9	69±10
**Men,** *n* (%)	91 (81)	100 (81)	95 (77)	286 (79)	111 (71)	103 (73)	102 (71)	316 (72)
**Baseline eGFR values,**[a] mL/min per 1.73 m^2, mean±SD	51±16	54±18	53±16	53±17	56±17	55±18	55±17	55±17
**UACR,** mg/g, median (IQR)	645 (336–1003)	738 (292–1653	654 (362–1233)	663 (327–1291)	504 (256–981)	476 (292– 863)	517 (274–1062)	504 (273– 955)
**Severity of albuminuria,** *n* (%)								
<300 mg/g	21 (19)	31 (25)	26 (21)	78 (22)	51 (33)	37 (26)	39 (27)	127 (29)
300 to <1000 mg/g	63 (56)	41 (33)	60 (48)	164 (46)	64 (41)	71 (50)	61 (43)	196 (45)
≥ 1000 mg/g	28 (25)	50 (41)	38 (31)	116 (32)	38 (24)	28 (20)	37 (26)	103 (23)
**ASCVD,**[b] *n* (%)	23 (20)	24 (20)	30 (24)	77 (21)	52 (34)	47 (33)	48 (34)	147 (34)
**Weight,** kg, mean±SD	68.8±12.7	71.6±14.2	70.6±12.7	70.4±13.2	93.3±21.1	90.9±20.5	89.7±20.0	91.4±20.6
**Height,** cm, mean±SD	163.2±8.3	164.4±7.6	163.9±8.9	163.9±8.3	168.9±9.9	169.5±10.0	168.7±9.7	169.0±9.8
**Body mass index,** kg/m^2, mean±SD	25.8±3.8	26.4±4.2	26.3±4.1	26.2±4.0	32.7±6.9	31.4±5.9	31.4±5.9	31.8±6.3

**Systolic blood pressure,** mmHg, mean±SD	132.7±14.0	132.4±11.9	133.3±12.0	132.8±12.6	136.7±13.3	137.4±14.7	137.1±12.9	137.1±13.6
**Serum potassium value,** mmol/L, mean±SD	4.5±0.4	4.5±0.5	4.6±0.4	4.5±0.4	4.4±0.4	4.5±0.5	4.5±0.4	4.5±0.4
**HbA1c,** %, mean±SD	7.2±1.2	7.3±1.3	7.3±1.2	7.3±1.2	7.4 ± 1.2	7.3 ±1.2	7.3 ± 1.2	7.3 ± 1.2
**Concomitant medications,** *n* (%)								
Insulin	25 (22)	31 (25)	42 (34)	98 (27)	83 (53)	65 (46)	71 (50)	219 (50)
Metformin	70 (62)	78 (63)	78 (63)	226 (63)	96 (62)	84 (60)	83 (58)	263 (60)
GLP-1 RA	15 (13)	11 (9)	14 (11)	40 (11)	53 (34)	41 (29)	48 (34)	142 (32)
DPP4 inhibitors	64 (57)	70 (57)	70 (57)	204 (57)	19 (12)	17 (12)	20 (14)	56 (13)
Insulin secretagogues	57 (50)	48 (39)	32 (26)	137 (38)	22 (14)	31 (22)	24 (17)	77 (18)
ACEis or ARBs	113 (100)	122 (99)	121 (98)	356 (99)	154 (99)	138 (98)	139 (97)	431 (98)
Antihypertensives	113 (100)	122 (99)	122 (98)	357 (99)	154 (99)	140 (99)	140 (98)	434 (99)
Beta-blockers	29 (26)	37 (30)	27 (22)	93 (26)	64 (41)	59 (42)	65 (46)	188 (43)
Calcium channel blockers	81 (72)	82 (67)	80 (65)	243 (68)	86 (55)	88 (62)	75 (52)	249 (57)
Diuretics	26 (23)	31 (25)	17 (14)	74 (21)	82 (53)	66 (47)	67 (47)	215 (49)
Antiplatelets	36 (32)	37 (30)	35 (28)	108 (30)	65 (42)	72 (51)	76 (53)	213 (48)
Statins	75 (66)	81 (66)	72 (58)	228 (63)	134 (86)	116 (82)	119 (83)	369 (84)

ACEi, angiotensin-converting enzyme inhibitor; ARB, angiotensin receptor blocker; ASCVD, atherosclerotic cardiovascular disease; DPP4, dipeptidyl peptidase-4; eGFR, estimated glomerular filtration rate; GLP-1 RA, glucagon-like peptide-1 receptor agonist; HbA1c, glycated hemoglobin; IQR, interquartile range; ROW, rest of world; SD, standard deviation; UACR, urinary albumin-to-creatinine ratio.

[a]The eGFR was calculated with the use of the Chronic Kidney Disease Epidemiology Collaboration equation, which was modified for the Japanese participants.

[b]Coded using MedDRA version 27.0.

Supplemental Figure 1. Least-squares mean change from baseline in serum potassium level over time in participants from Asia (A) and Europe/North America (B).

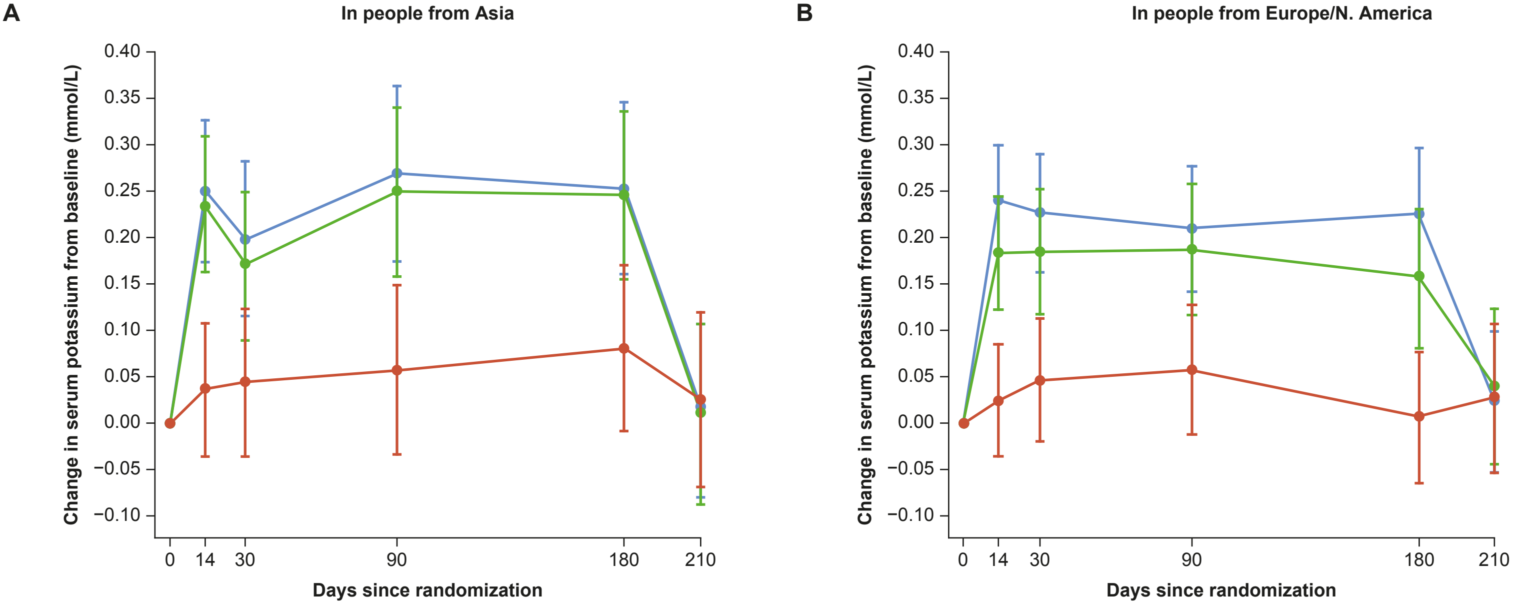

No. of patients (A: In people from Asia)

	0	14	30	90	180	210
Finerenone + empagliflozin	113	107	108	108	106	106
Finerenone	123	116	118	115	113	110
Empagliflozin	124	121	118	115	112	114

No. of patients (B: In people from Europe/N. America)

	0	14	30	90	180	210
Finerenone + empagliflozin	154	146	153	146	138	147
Finerenone	141	134	134	127	127	125
Empagliflozin	142	139	136	135	132	131

Finerenone + empagliflozin — Finerenone — Empagliflozin

Supplemental Figure 2. Least-squares mean change from baseline in systolic blood pressure over time in participants from Asia (A) and Europe/North America (B).

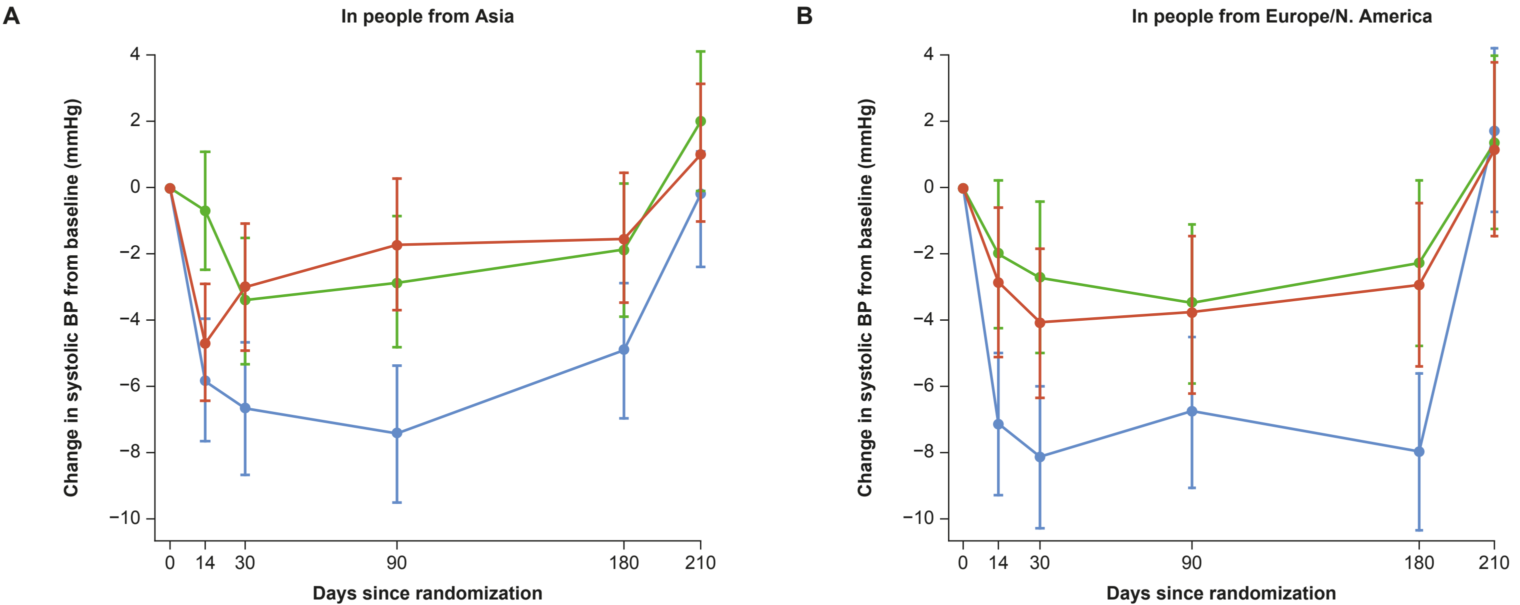

No. of patients (A)

	0	14	30	90	180	210
Finerenone + empagliflozin	113	107	109	109	107	108
Finerenone	123	119	121	118	116	114
Empagliflozin	124	122	120	118	116	117

No. of patients (B)

	0	14	30	90	180	210
Finerenone + empagliflozin	155	148	153	147	140	145
Finerenone	141	138	135	130	128	129
Empagliflozin	142	139	139	135	131	131

Finerenone + empagliflozin   Finerenone   Empagliflozin

Supplemental Figure 3. Least-squares mean change from baseline in eGFR over time in participants from Asia (A) and Europe/North America (B).

eGFR, estimated glomerular filtration rate

[a]The eGFR was calculated with the use of the Chronic Kidney Disease Epidemiology Collaboration equation, which was modified for the Japanese participants.

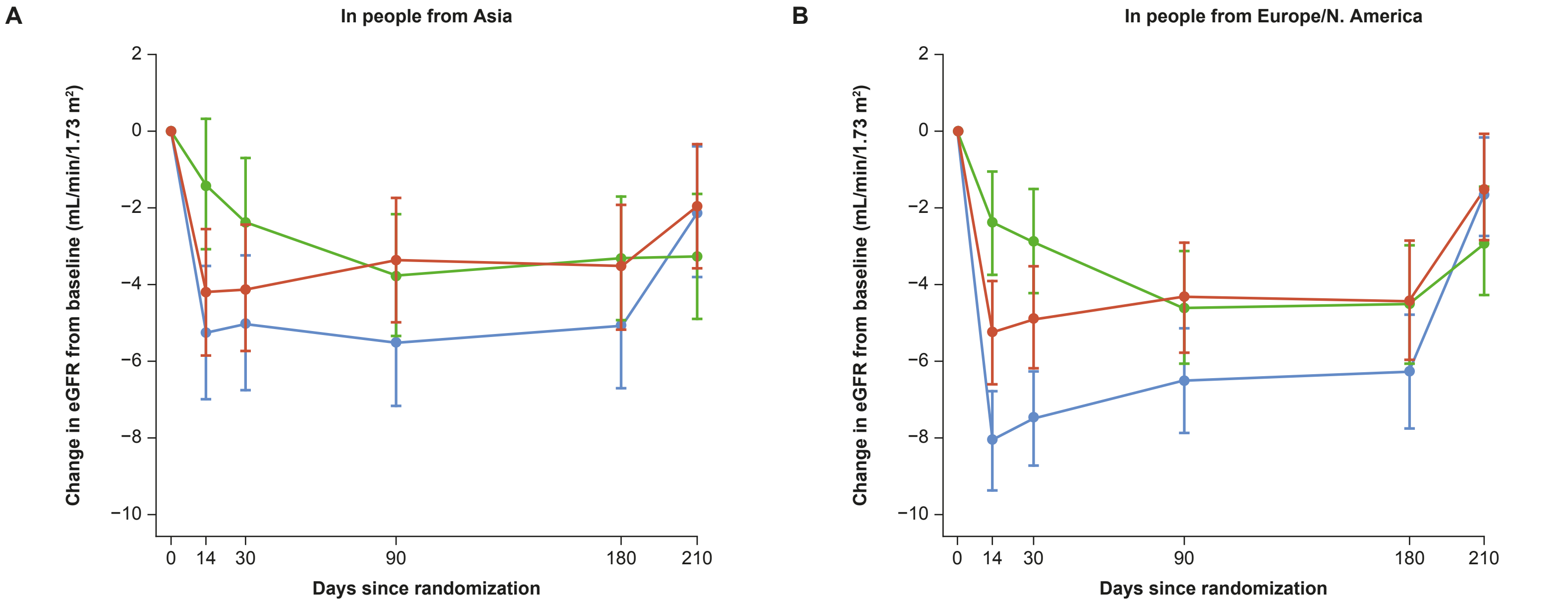

No. of patients

Finerenone + empagliflozin	113	107	109	109	107	108
Finerenone	123	116	119	117	114	111
Empagliflozin	124	122	120	117	113	115

No. of patients

Finerenone + empagliflozin	156	146	152	145	136	145
Finerenone	139	134	132	126	125	123
Empagliflozin	141	136	135	132	129	128

Finerenone + empagliflozin — Finerenone — Empagliflozin
